# Intestinal Epithelial Cell-Intrinsic Deletion of *Setd7* Identifies Role for Developmental Pathways in Immunity to Helminth Infection

**DOI:** 10.1371/journal.ppat.1005876

**Published:** 2016-09-06

**Authors:** Menno J. Oudhoff, Frann Antignano, Alistair L. Chenery, Kyle Burrows, Stephen A. Redpath, Mitchell J. Braam, Georgia Perona-Wright, Colby Zaph

**Affiliations:** 1 The Biomedical Research Centre, University of British Columbia, Vancouver, British Columbia, Canada; 2 Center of Molecular Inflammation Research, Department of Cancer Research and Molecular Medicine, Norwegian University of Science and Technology, Trondheim, Norway; 3 Department of Microbiology and Immunology, Life Sciences Institute, University of British Columbia, Vancouver, British Columbia, Canada; 4 Department of Pathology and Laboratory Medicine, University of British Columbia, Vancouver, British Columbia, Canada; 5 Infection and Immunity Program, Monash Biomedicine Discovery Institute and Department of Biochemistry and Molecular Biology, Monash University, Clayton, Victoria, Australia; New York University, UNITED STATES

## Abstract

The intestine is a common site for a variety of pathogenic infections. Helminth infections continue to be major causes of disease worldwide, and are a significant burden on health care systems. Lysine methyltransferases are part of a family of novel attractive targets for drug discovery. SETD7 is a member of the Suppressor of variegation 3-9-Enhancer of zeste-Trithorax (SET) domain-containing family of lysine methyltransferases, and has been shown to methylate and alter the function of a wide variety of proteins *in vitro*. A few of these putative methylation targets have been shown to be important in resistance against pathogens. We therefore sought to study the role of SETD7 during parasitic infections. We find that *Setd7* ^-/-^ mice display increased resistance to infection with the helminth *Trichuris muris* but not *Heligmosomoides polygyrus bakeri*. Resistance to *T*. *muris* relies on an appropriate type 2 immune response that in turn prompts intestinal epithelial cells (IECs) to alter differentiation and proliferation kinetics. Here we show that SETD7 does not affect immune cell responses during infection. Instead, we found that IEC-specific deletion of *Setd7* renders mice resistant to *T*. *muris* by controlling IEC turnover, an important aspect of anti-helminth immune responses. We further show that SETD7 controls IEC turnover by modulating developmental signaling pathways such as Hippo/YAP and Wnt/β-Catenin. We show that the Hippo pathway specifically is relevant during *T*. *muris* infection as verteporfin (a YAP inhibitor) treated mice became susceptible to *T*. *muris*. We conclude that SETD7 plays an important role in IEC biology during infection.

## Author Summary

The gastrointestinal tract is a common site for infection by a variety of pathogens. For example, gut-dwelling parasitic worms currently infect over a billion people, mostly in developing nations. Deworming strategies have been shown to improve physical and intellectual development of infected children, but current therapies do not offer a sustainable solution. We still have too little insight into how these pathogens are causing disease and how immunity to them is regulated. In this study we show that SETD7, an enzyme that modifies the function of other proteins by methylation, plays an important role in the development of intestinal immunity to the helminth parasite *Trichuris muris* but not *Heligmosomoides polygyrus bakeri*. Specifically, we show that SETD7 affects intestinal epithelial turnover, a key mechanism through which *T*. *muris* worms are extruded from the body. Our studies identify pathways that are important for immunity to infection, that were previously believed to be involved primarily during embryonic development.

## Introduction

The gastrointestinal tract is responsible for absorption of nutrients and water, but at the same time it has an important role in acting as a barrier to the external environment [1]. This barrier function is further complicated by the requirement to respond appropriately to pathogens, but remain tolerant to innocuous antigens like commensal organisms and food. Understanding the molecular pathways that control intestinal homeostasis is critical for promoting immunity and limiting inflammation.

Intestinal homeostasis is the result of a complex interplay between the environment, intestinal epithelial cells (IECs), mesenchymal cells, vascular endothelial cells, and cells of the innate and adaptive immune systems. This interconnected system relies on a multitude of signaling pathways in the various cell types, and aberrant signaling is a key feature in chronic intestinal inflammatory diseases [2]. However, for immunity against certain pathogens, a temporally controlled high level of immune activation is required, including strong inflammatory cues that may lead to significant tissue damage [3–6]. A repair process is then initiated that is essential to regain barrier function and prevent sustained inflammation. IECs play an important role in many of these processes as they can act as the first sensor of pathogens [7], they execute immune responses by responding to specific cues [8], and initiate repair processes that require intestinal stem cells (ISCs) [9–11]. Despite their importance, the molecular pathways that regulate IEC function in immunity, inflammation and repair remain poorly described.

IECs have a remarkable turnover of around 3–5 days [12]. During homeostasis this turnover is driven by ISCs that reside at the bottom of crypts and divide every day [13]. Upon division ISCs leave the stem cell zone to become progenitors for either enterocytes or one of the secretory lineages such as goblet cells and Paneth cells [12]. A variety of signal transduction pathways, including Wnt, Notch, and Hippo are important regulators of ISC and IEC biology. Although several studies have emerged identifying the importance of IECs in immunity to pathogens [6,8,14,15], the molecular pathways that control IEC dynamics during infection remain unknown.

Lysine methyltransferases represent part of a family of novel druggable targets that are currently being investigated for a variety of diseases [16]. SETD7 is a member of the Suppressor of variegation 3-9-Enhancer of zeste-Trithorax (SET) domain-containing family of lysine methyltransferases, and has been shown to methylate and alter the function of a wide variety of proteins *in vitro* [17]. SETD7 has been shown to have *in vitro* effects on a wide variety of signaling intermediates including NF-κB and STAT3 [18,19] that are crucial for immunity to pathogens [6,8]. We have recently found that there is an interplay between SETD7 and the Hippo and Wnt pathways, which are evolutionarily conserved signaling pathways that are important for IEC homeostasis, regeneration and tumorigenesis [20–22]. In this study we identify a critical role for IEC-intrinsic expression of *Setd7* in immunity to helminth infection.

## Results

### *Setd7* ^-/-^ mice are more resistant to *T*. *muris* infection

Immunity to infection with the intestinal helminth parasite *T*. *muris* is mediated by a complex interplay between IECs and the innate and adaptive immune systems [5,8,23]. Commonly, resistance relies on the development of an adaptive T_H_2 cell response as opposed to a non-protective T_H_1 cell response that leads to susceptibility. This T_H_2 cell response is mediated by a wide variety of innate and adaptive immune cells that require appropriate signaling [5].

**Fig 1:**
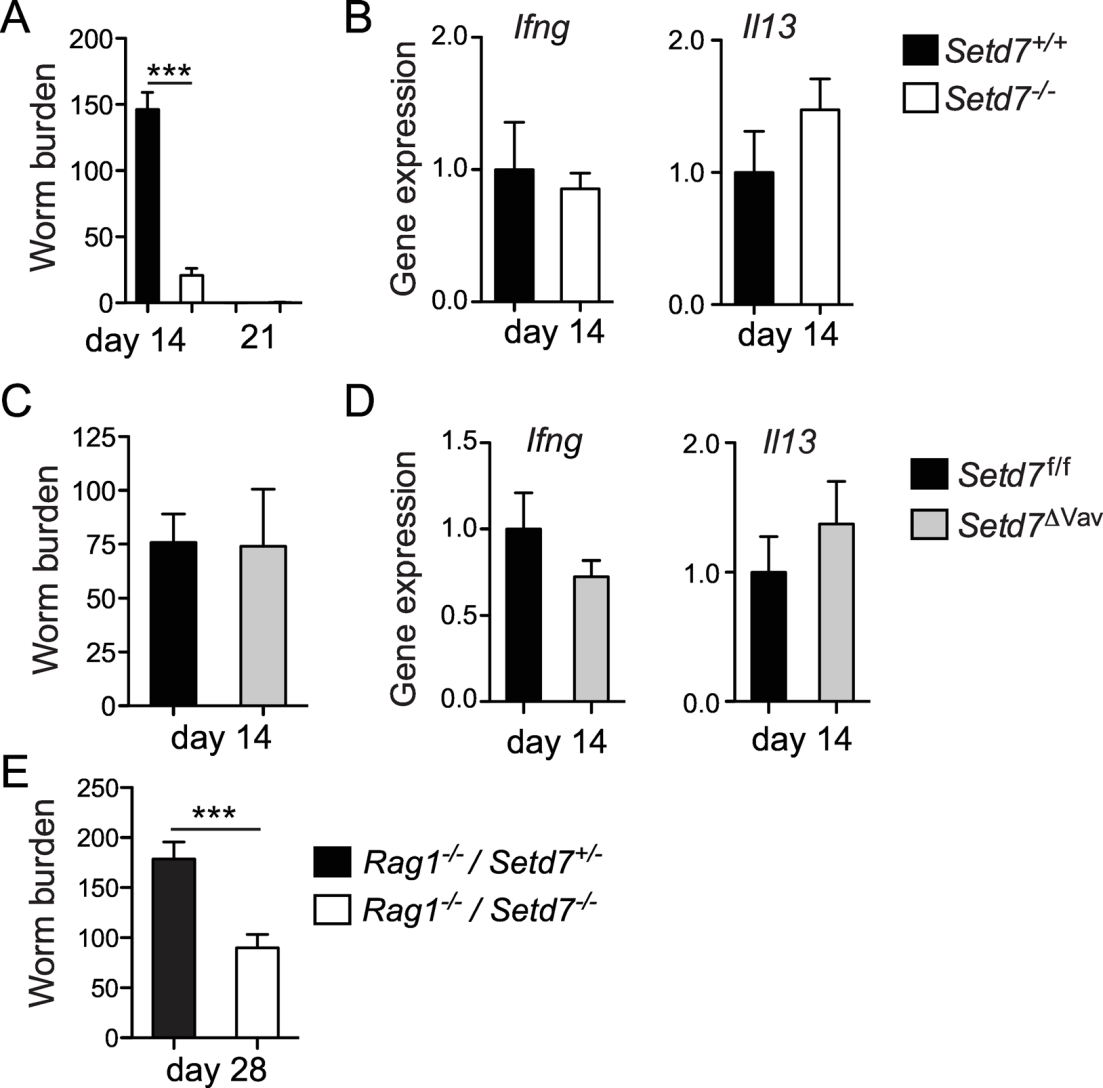
Deletion of *Setd7* renders mice resistant to *T*. *muris*. (A & C) Indicated mice were infected with 200 *T*. *muris* eggs and killed on day 14 or day 21 after infection. Worm burdens were determined microscopically. (n = 5, *** P<0.001). (B & D) *Ifng* and *Il13* mRNA expression of proximal colon tissue was assessed by qPCR at day 14 post infection. Gene expression is relative to infected control mice (*Setd7* ^+/+^ and *Setd7* ^f/f^ for B and D respectively) (n = 5) (E) Indicated mice were infected with 200 *T*. *muris* eggs and killed on day 28 post infection. Worm burdens were determined microscopically. (n≥9, *** P<0.001).

To test whether *Setd7* plays a role in the development of intestinal immunity, we infected *Setd7* ^+/+^ and *Setd7* ^-/-^ mice with 200 embryonated *T*. *muris* eggs [24]. *Setd7* ^-/-^ mice display no overt developmental phenotypes and we did not observe compensational expression of related lysine methyltransferases in the intestine upon deletion of *Setd7* (S1 Fig). At day 21 post infection both wild type and knock out mice had fully cleared the infection, indicating that complete loss of *Setd7* does not render mice susceptible to *T*. *muris* infection (Fig 1A). In contrast, at day 14 we found that although *Setd7* ^*+/+*^ mice were in the midst of expelling the worms, *Setd7* ^-/-^ mice had already cleared most of their worm burden (Fig 1A), suggesting that *Setd7* ^-/-^ mice were more resistant to *T*. *muris* infection. Enhanced resistance to infection with *T*. *muris* was not due to an intrinsic difference in microbiota-mediated hatching [25], as fecal contents from either strain of mice were equally able to induce egg hatching *in vitro* (S2A Fig) and we detected equal worm burdens at day 12 post infection (S2B Fig). Despite the increased immunity to infection in *Setd7* ^-/-^ mice, we did not detect any significant differences in expression of T_H_1 cell- or T_H_2 cell-mediated cytokine genes such as *Ifng* and *Il13* in the gut by qPCR (Figs 1B and S2C). These results suggest that the enhanced resistance to infection in SETD7-deficient mice was neither due to an increased protective type 2 nor a decreased non-protective type 1 immune response. Consistent with this hypothesis, infection of mice with a hematopoietic cell-intrinsic deletion of *Setd7* (*Setd7* ^ΔVav^ mice, generated by crossing *Setd7* ^f/f^ mice with *Vav-*Cre mice) revealed that SETD7-deficiency in immune cells had no effect on resistance to infection. At day 14 post-infection, both *Setd7* ^f/f^ and *Setd7* ^ΔVav^ mice had equal worm burdens (Fig 1C) and similar gene expression levels of *Ifng* and *Il13* in the intestine (Fig 1D), suggesting that SETD7 expression in hematopoietic cells was not responsible for the increased immunity to *T*. *muris*. Importantly, *Setd7* ^-/-^ mice that lack an adaptive immune system (*Rag1* ^-/-^ / *Setd7* ^-/-^ mice, generated by crossing *Setd7* ^-/-^ mice with *Rag1* ^-/-^ mice) also displayed increased resistance to infection with *T*. *muris* compared to *Rag1* ^-/-^ / *Setd7* ^+/-^ littermate controls, with a significant reduction in worm burden at day 28 post-infection (Fig 1E). Thus, in the absence of SETD7, an adaptive immune system is dispensable for the development of immunity to *T*. *muris*, suggesting that SETD7 expression in non-hematopoietic cells is a critical component of the response to *T*. *muris* infection.

### IEC-intrinsic deletion of *Setd7* in mice leads to increased resistance to *T*. *muris* infection

**Fig 2:**
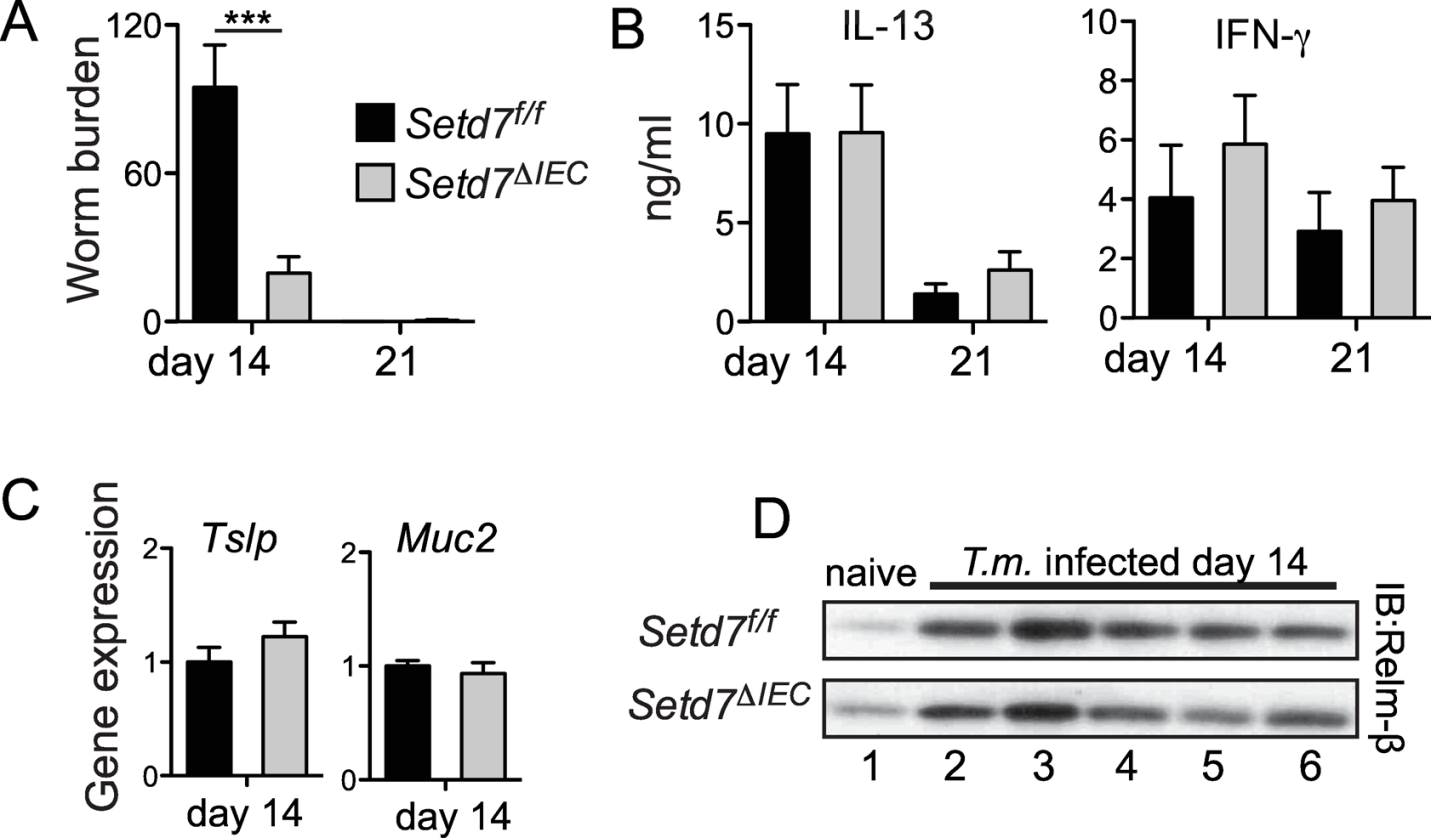
Deletion of *Setd7* specifically in IECs renders mice resistant to *T*. *muris*. (A) *Setd7* ^f/f^ (black bars) and *Setd7* ^ΔIEC^ (grey bars) mice were infected with 200 *T*. *muris* eggs and worm burdens were determined at day 14 or day 21 post infection (n≥9 for day 14, n = 6 for day 21, *** P<0.001). (B) Mesenteric lymph node cells from infected mice were re-stimulated for 72 h. IL-13 and IFN-γ concentrations in supernatants was determined by ELISA. (n≥4). (C) Expression of *Tslp* and *Muc2* in proximal colon at day 14 post infection with *T*. *muris* was assessed by qPCR. Expression is relative to infected control (*Setd7* ^f/f^) mice. (n≥8). (D) Protein levels of RELMβ that was secreted into the gut lumen of naïve and *T*. *muris* infected mice evaluated by Western blot.

Several studies have shown that immunity to *T*. *muris* is associated with a variety of IEC responses, including goblet cell hyperplasia and mucin production, expression of secreted molecules such as thymic stromal lymphopoietin (TSLP) and resistin-like molecule-β (RELMβ), and increased proliferation and turnover [8,15,26–28]. We next directly tested whether IEC-intrinsic deletion of *Setd7* would lead to increased resistance to *T*. *muris* infection. We infected mice with an IEC-specific deletion of Setd7 (*Setd7* ^ΔIEC^ mice) [20] with *T*. *muris* and found that similar to *Setd7* ^-/-^ mice, *Setd7* ^ΔIEC^ mice displayed enhanced resistance to infection, with reduced worm burden at day 14 post-infection (Fig 2A). We failed to observe any differences in levels of IFN-γ or IL-13 produced by restimulated mesenteric lymph node (mLN) cells (Fig 2B) or in gene expression by qPCR of intestinal tissues (S3A Fig) between *Setd7* ^f/f^ and *Setd7* ^ΔIEC^ mice. Further, we detected equal worm burdens and *Ifng* and *Il13* expression between infected *Setd7* ^f/f^ and *Setd7* ^ΔIEC^ mice at day 12 post infection (S3B and S3C Fig). Thus, IEC-intrinsic expression of SETD7 negatively regulates resistance to infection with *T*. *muris*.

IECs have been shown to differentiate into goblet cells following infection with *T*. *muris* in response to T_H_2 cell-derived cytokines and produce effector molecules such as the mucins Muc5AC [28] and Muc2 [27], cytokines such as TSLP [29] as well as the small protein RELMβ [26]. Consistent with our results demonstrating equivalent T_H_2 cell responses, we did not observe any differences in expression of *Muc5ac* (S3D Fig), *Muc2* or *Tslp* (Fig 2C) or secretion of RELMβ into the intestinal lumen (Fig 2D) between *Setd7* ^*f/f*^ and *Setd7* ^ΔIEC^ mice following *T*. *muris* infection. Thus, the increased resistance to *T*. *muris* in *Setd7* ^ΔIEC^ mice is not due to the enhanced production of effector molecules by IECs.

### Crypt length and IEC turnover dictate resistance to *T*. *muris* in *Setd7* ^ΔIEC^ mice

**Fig 3:**
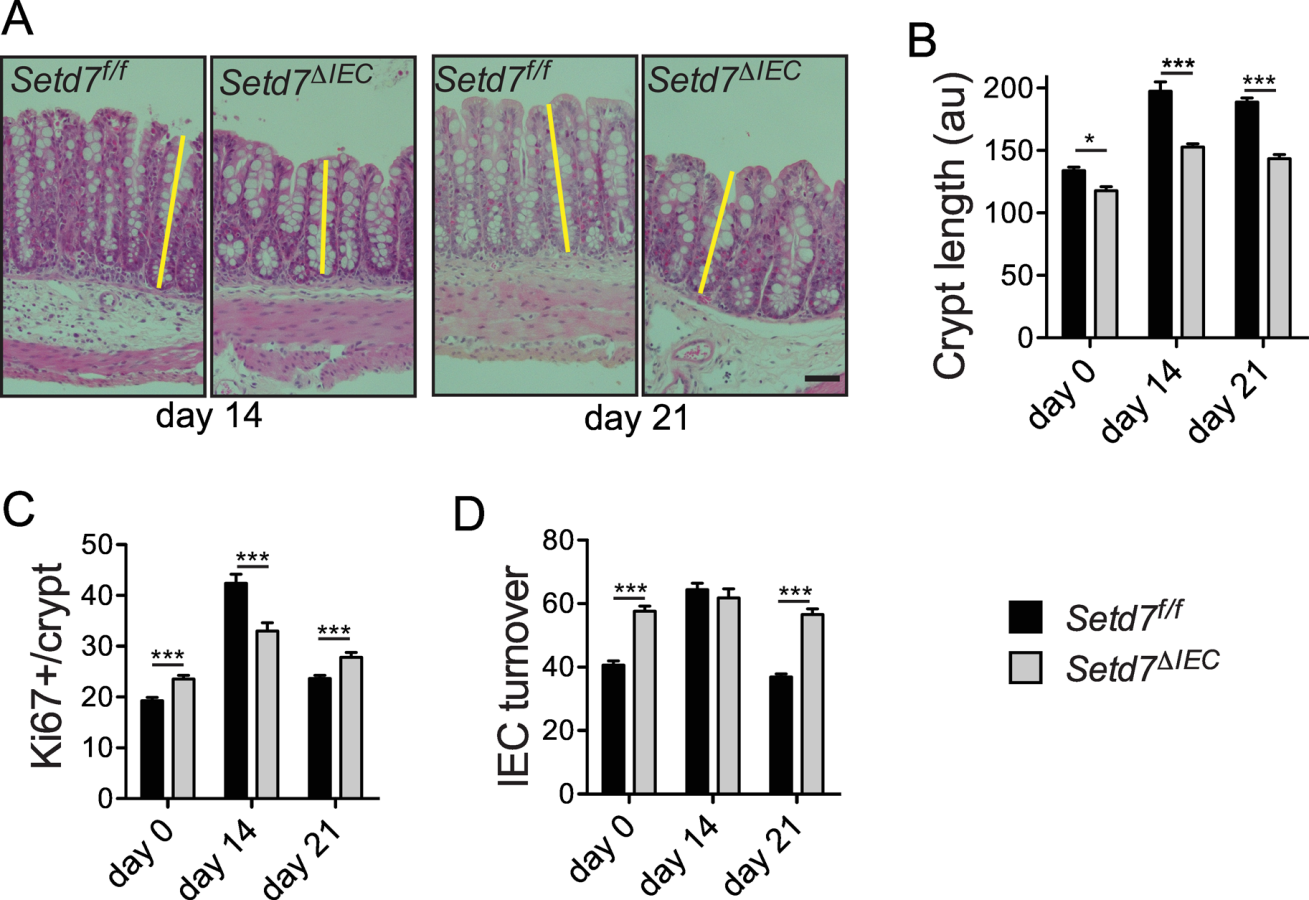
*Setd7* regulates IEC turnover responses after *T*. *muris* infection. (A) H&E stained caecal sections of *Setd7* ^f/f^ and *Setd7* ^ΔIEC^ mice at day 14 and day 21 post infection with *T*. *muris*. Yellow lines indicate crypt length. Original magnification is 100X. Bar = 50 μm (B) Crypt length (au = arbitrary units) of caecums of naïve (day 0) and *T*. *muris* infected mice (day 14 and day 21 post infection). (n≥30 of at least 4 mice, * P<0.05, *** P<0.001). (C) Proliferation as measured by counting Ki67+ cells per crypt from images such as in S4A. (n≥20 of at least 4 mice, *** P<0.001). (D) IEC turnover was determined by dividing Ki67+ cells by total DAPI+ cells from images such as shown in S4A. Of note, crypt length correlated with total DAPI+ cells per crypt at all time points (see S4C). (n≥20 of at least 4 mice, *** P<0.001).

In addition to IEC differentiation, it has been shown that IEC turnover is important for resistance to infection with *T*. *muris* [15]. IEC turnover can be defined by combining crypt length (or cells per crypt) with the number of proliferating cells [15,28]. For example, short crypts with many proliferating cells have high turnover whereas long crypts with few proliferating cells have slow turnover. In resistant mice, IEC turnover is induced to clear *T*. *muris*, whereas in susceptible mice this increased turnover does not occur [15]. First, we carefully analyzed crypt length and found that *Setd7* ^ΔIEC^ mice have shorter crypts compared to *Setd7* ^f/f^ mice before and throughout *T*. *muris* infection (Fig 3A and 3B) [20]. Crypt length was highly correlated with the number of cells per crypt as counted by DAPI (S4A–S4C Fig). Next, we analyzed the number of IECs that are proliferating by enumerating Ki67^+^ cells per crypt (Fig 3C and S4A Fig). We found that prior to infection (day 0) an increased number of IECs from *Setd7* ^ΔIEC^ mice stained positive for Ki67, suggesting increased proliferation, consistent with our previous study [20]. To measure IEC turnover we calculated the frequency of cells proliferating by dividing the number of Ki67^+^ cells by the DAPI^+^ (total number) cell numbers (Fig 3D). Combining proliferation with crypt length (or number of cells per crypt) is essential because increased proliferation does not always correlate with turnover, *i*.*e*. it may just lead to longer crypts. We found that under homeostatic conditions, *Setd7* ^ΔIEC^ mice display faster IEC turnover compared to *Setd7* ^f/f^ mice (Fig 3D). Following infection with *T*. *muris*, *Setd7* ^f/f^ mice increase IEC turnover (Fig 3D)[15], suggesting that this is the peak turnover that is required for parasite expulsion. Our results suggest that the turnover induced by *T*. *muris* in *Setd7* ^f/f^ mice that is associated with worm expulsion is already present during homeostasis in naive *Setd7* ^ΔIEC^ mice, resulting in enhanced worm expulsion. Thus, loss of SETD7 is associated with increased IEC turnover that promotes resistance to *T*. *muris*.

### IEC-specific deletion of *Setd7* renders mice resistant to chronic *T*. *muris* infection

**Fig 4:**
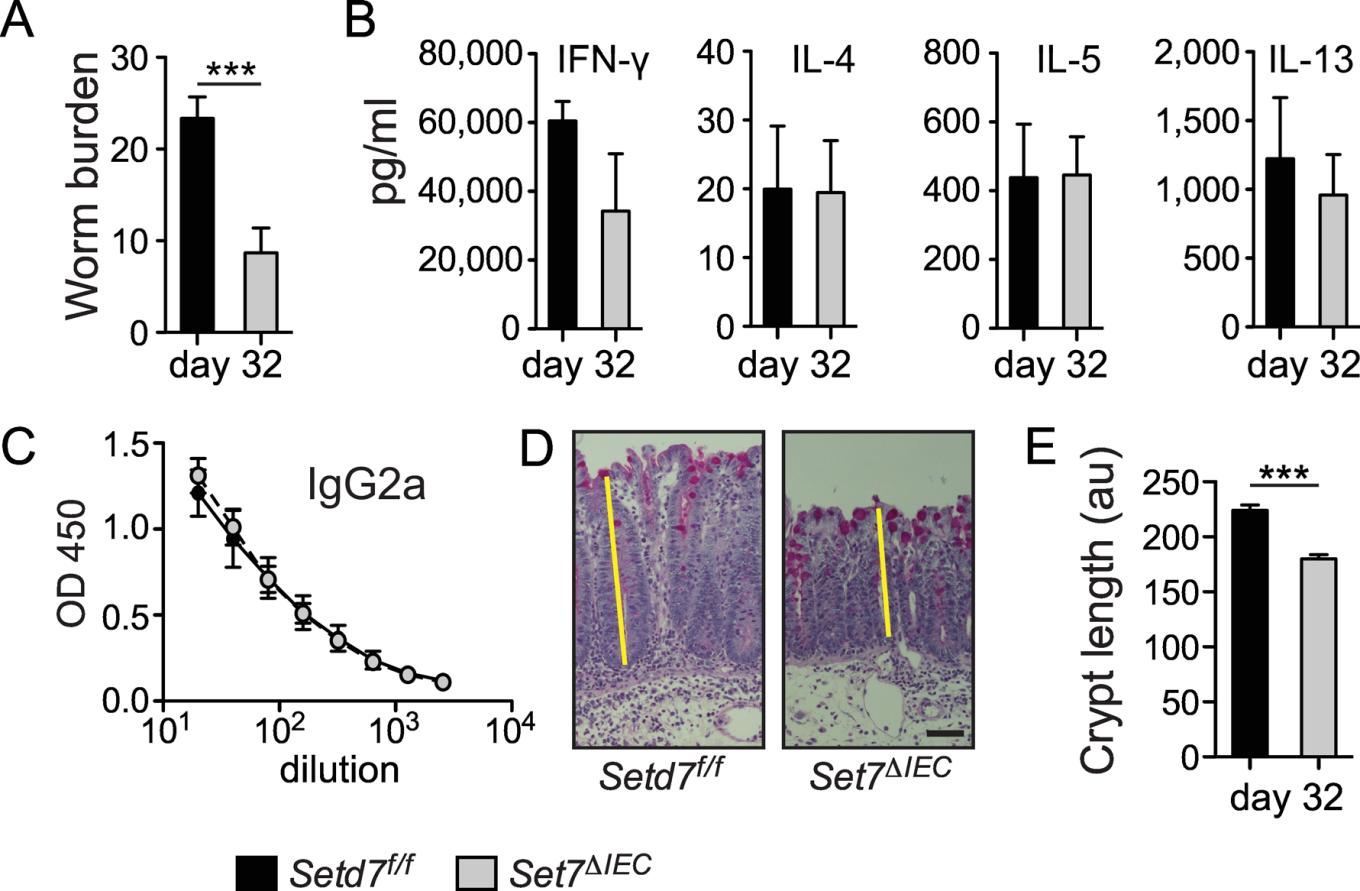
*Setd7* ^ΔIEC^ mice resistant to low dose *T*. *muris* infection. (A) *Setd7* ^f/f^ and *Setd7* ^ΔIEC^ mice were infected with ~35 *T*. *muris* eggs (low dose) and worm burdens were determined at day 32 post infection (n = 9, pooled from 2 independent experiments, *** P<0.001). (B) Cytokine production of mesentyric lymph node cells that were re-stimulated for 72 h was determined by ELISA. (n≥4). (C) Serially diluted serum of infected mice was analyzed by ELISA to measure *T*. *muris-*specific IgG2a. (n≥4). (D) Periodic acid-Schiff stained caecal sections show a robust depletion of goblet cells in both *Setd7* ^f/f^ and *Setd7* ^ΔIEC^ mice at day 32 post infection. Yellow lines indicate crypt length. Original magnification is 100X. Bar = 50 μm (E) Crypt length (au = arbitrary units) in caecums of mice at day 32 post infection. (n≥60 of 9 mice in each group, *** P<0.001). *Setd7* ^f/f^ black bars, *Setd7* ^ΔIEC^ grey bars.

Although a high-dose *T*. *muris* infection (>100 eggs) is very suitable for studying various immunological processes in mice, it is arguably not the perfect model for studying chronic helminth infections that dramatically affect human lives, mainly in developing countries [30]. In contrast, a low-dose (<50 eggs) *T*. *muris* infection leads to a persistent infection with a chronic worm burden [31]. To test if SETD7 played a role in the development of chronic helminth infection, we infected mice with a low-dose (~30 eggs) of *T*. *muris* infection. We found that *Setd7* ^ΔIEC^ mice were also more resistant to chronic infection with *T*. *muris* compared to *Setd7* ^f/f^ mice at day 32 post infection (Fig 4A). At day 21 post infection we observed equivalent infection rates between *Setd7* ^f/f^ and *Setd7* ^ΔIEC^ mice, which was associated with equivalent expression of *Ifng* and *Il13* in the intestine (S5A and S5B Fig). Further, we did not observe differences in adaptive immune responses as measured by cytokine secretion following polyclonal restimulation of mLN cells, serum levels of *T*. *muris*-specific IgG2a and IgG1, as well as cytokine gene expression in the intestinal tissue at day 32 post infection (Fig 4B and 4C and S5C and S5D Fig). However, consistent with our results with high-dose infection, we did observe differences in crypt length between *Setd7* ^f/f^ and *Setd7* ^ΔIEC^ mice, albeit with equal robust reduction of goblet cells (Fig 4D and 4E). Thus, loss of SETD7 in IECs leads to increased resistance to chronic helminth infection independent of the immune response, identifying a potential new therapeutic target to treat persistent helminth infections.

### IEC-specific deletion of *Setd7* does not modify resistance to *Heligmosomoides polygyrus bakeri*

**Fig 5:**
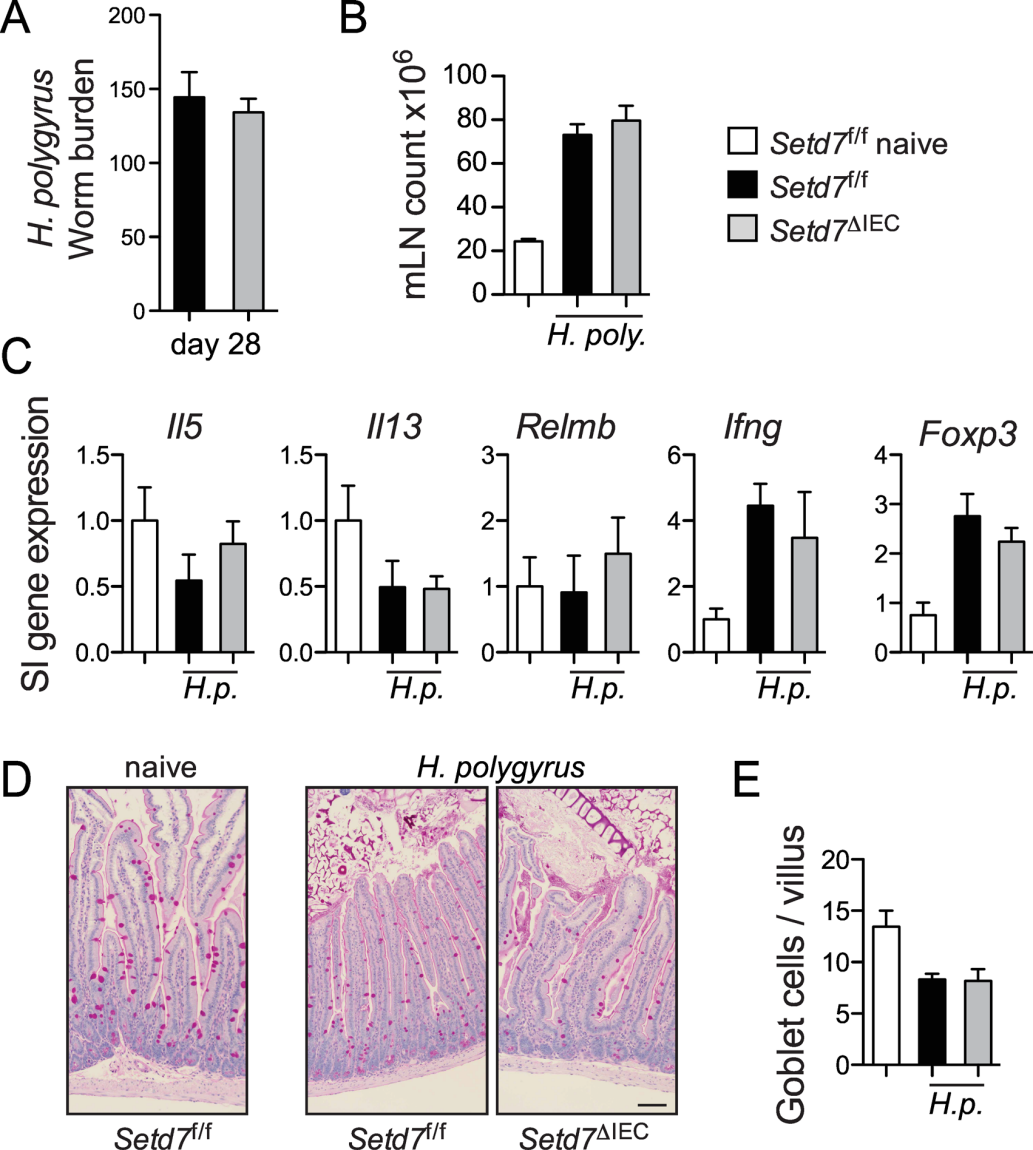
*Setd7* ^ΔIEC^ mice do not have increased resistance against infection with *H*. *p*. *bakeri*. (A) *Setd7* ^f/f^ and *Setd7* ^ΔIEC^ mice were infected with ~ 200 *H*. *p*. *bakeri* eggs and were killed day 28 post infection. Worm burdens were determined microscopically from the small intestine. (B) Mesenteric lymph node (mLN) cell counts from naïve and *H*. *p*. *bakeri* infected mice (day 28 post infection). (C) Gene expression of indicated genes in small intestine (SI) that was adjacent to infection site at day 28 post infection with *H*. *p*. *bakeri*. Expression is relative to naïve mice (white bars). (D) Periodic acid-Schiff (PAS) staining of small intestinal tissue sections of indicated mice at day 28 post infection. Bar = 100 μm. (E) PAS+ cells per villus were counted from images as shown in (D). (A-E) n = 3 (naives), n = 8 (infected) from 2 independent experiments. *Setd7* ^f/f^ black bars, *Setd7* ^ΔIEC^ grey bars.

*T*. *muris* resides in the caecum embedded in the intestinal epithelium in close association with IECs [32]. IEC proliferation and turnover has been shown to play a role in expulsion via an ‘epithelial escalator’ [15]. In contrast, larvae of *Heligmosomoides polygyrus bakeri* penetrate the epithelium into the submucosa, undergo two molts, and the worms resurface in the lumen wrapped around small intestinal villi [33]. Resistance to *H*. *p*. *bakeri* is accomplished by a T_H_2-cell-mediated ‘weep and sweep’ response, which consists of goblet-cell-mediated secretion of mucins (weep) and muscle contraction (sweep) [34]. To test whether *Setd7* ^ΔIEC^ mice also have altered resistance against *H*. *p*. *bakeri*, we infected *Setd7* ^f/f^ and *Setd7* ^ΔIEC^ mice with ~200 larvae. At 28 days post infection we found no difference in worm burden or induction of mesenteric lymph node cell numbers (Fig 5A and 5B). There was an upregulation of expression of indicators of T_H_1 (*Ifng*) and T_reg_ (*Foxp3*) cell responses at this time point (Fig 5C). However, we failed to observe any differences in expression of *Il5*, *Il13*, *Relmb*, *Ifng*, *Foxp3* between infected *Setd7* ^f/f^ and *Setd7* ^ΔIEC^ mice (Fig 5C). We observed a reduction of periodic acid-Schiff (PAS) positive cells in infected animals compared to naïve controls, but this too was equivalent between *Setd7* ^f/f^ and *Setd7* ^ΔIEC^ mice (Fig 5D and 5E). Thus, in contrast to *T*. *muris*, IEC-intrinsic deletion of *Setd7* does not affect immunity against *H*. *p*. *bakeri*.

### SETD7 regulates Wnt and Hippo signaling during *T*. *muris* infection

**Fig 6:**
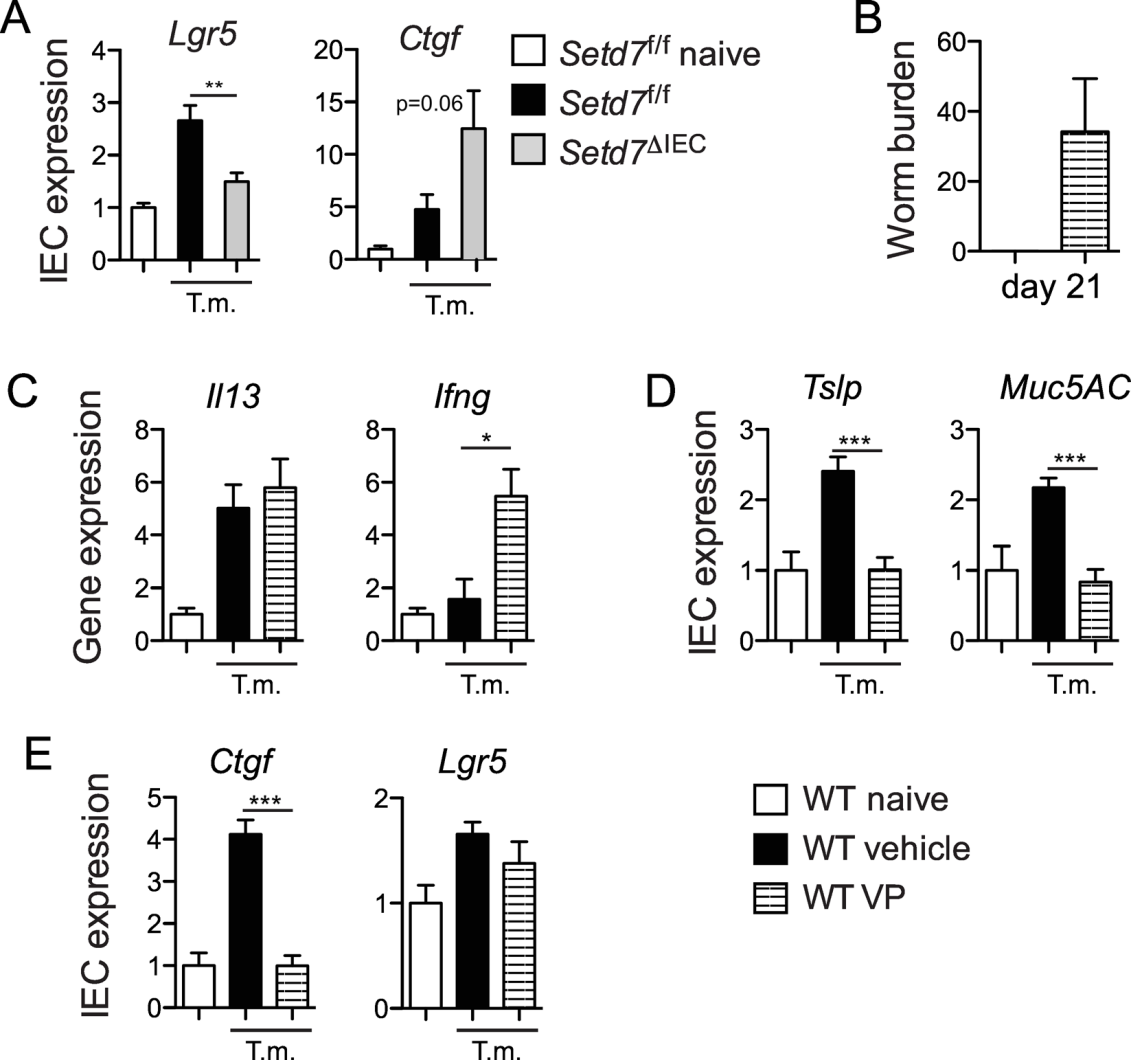
Inhibition of YAP-TEAD interactions *in vivo* in resistance to *T*. *muris*. (A) IEC-specific expression of Wnt (*Lgr5*) and Hippo (*Ctgf*) target genes in *T*. *muris* infected *Setd7* ^f/f^ (black bars) and *Setd7* ^ΔIEC^ (grey bars) mice. Expression is relative to naïve mice (white bars). (n≥8, ** P<0.01). (B) Worm burdens at day 21 post infection of wild type (WT) mice. Mice were either vehicle treated (black bars) or vertepofin (VP) treated (striped bars) (n≥6). (C) Gene expression of *Il13* and *Ifng* from proximal colon at day 21 post infection. Expression is relative to uninfected, untreated WT mice (white bars, WT naive). (n≥6, * P<0.05) (D & E) IEC-specific expression of effector (*Tslp* and *Muc5AC*) and Hippo and Wnt target (*Ctgf* and *Lgr5)* genes. Expression is relative to naïve mice (white bars). (n≥6, *** P<0.001).

We have previously shown that increased IEC proliferation and turnover in the absence of SETD7 during homeostasis correlated with dysregulated signaling by the Hippo pathway [20], a pathway well known to control IEC proliferation [35–38]. SETD7 is required for the proper subcellular localization of the Hippo pathway transducer YAP [20]. In the absence of SETD7, YAP is enriched in the nucleus where it interacts with the TEAD family of transcription factors, resulting in heightened YAP/TEAD-dependent gene expression. Recent studies have also linked Hippo/YAP signaling with Wnt/β-Catenin signaling in the control of IEC proliferation and turnover [38–41]. Indeed, we have recently shown that SETD7-dependent methylation of YAP is required for optimal Wnt/β-Catenin signaling during intestinal regeneration and tumorigenesis [22]. We therefore analyzed Wnt and Hippo activity in IECs during *T*. *muris* infection by measuring specific target genes of each pathway. We found that both Wnt (*Lgr5* and *Axin2*) and Hippo (*Ctgf* and *Gli2*) target genes are upregulated in IECs during *T*. *muris* infection of control *Setd7* ^f/f^ mice compared to naïve mice (Fig 6A and S6 Fig). In the absence of SETD7, we found that Wnt target gene expression is reduced while Hippo target gene expression is increased in IECs following infection (Fig 6A and S6 Fig). Thus, IEC-intrinsic expression of SETD7 regulates IEC turnover during *T*. *muris* infection, possibly by influencing Wnt and Hippo signaling.

### YAP inhibitor verteporfin renders mice susceptible to high-dose *T*. *muris* infection

We next tested whether blocking YAP transcriptional activity would affect resistance to infection with *T*. *muris*. To do this, we treated wild type mice with liposome-encapsulated verteporfin (VP), a recently discovered inhibitor of YAP-TEAD interactions [42]. Following infection with *T*. *muris*, we found that VP-treated mice failed to fully clear their infection by day 21 in comparison to vehicle-treated mice (Fig 6B). Consistent with the increased susceptibility to infection, we observed a significant increase in gene expression of the T_H_1 cell-associated cytokine *Ifng* in the intestine of VP-treated mice, even though *Il13* levels were equal (Fig 6C). Further, induction of IEC-specific genes associated with resistance to infection such as *Muc5ac* and *Tslp* was abrogated by VP treatment (Fig 6D). As expected, VP treatment abolished *T*. *muris*-mediated upregulation of YAP target gene *Ctgf* (Fig 6E), but we did not observe a striking effect on Wnt/β-Catenin target genes such as *Lgr5* (Fig 6E). Together, these experiments show that YAP-TEAD interactions are important for the development of immunity to *T*. *muris*.

## Discussion

In this study we describe the role of SETD7 during helminth infection. We find that deletion of *Setd7* in IECs renders mice resistant to the helminth *T*. *muris* but does not affect *H*. *p*. *bakeri* infection. Interestingly, *Setd7* does not affect T helper cell responses or goblet cell differentiation, both of which are deemed very important for resistance against *T*. *muris* [27,28,43,44]. Instead, we find that epithelial turnover is affected by the lack of *Setd7*, and therefore, IEC turnover can be dominant over adaptive immune responses in terms of importance for resistance to *T*. *muris*. In *Setd7* ^ΔIEC^ mice we observed higher turnover during homeostasis compared to *Setd7* ^f/f^ mice, demonstrating that SETD7 regulates IEC turnover independently of infection-induced immune cell cues. In contrast, control *Setd7* ^f/f^ mice relied on type 2 immune responses to increase its IEC turnover to expel *T*. *muris* worms. We quantified turnover by examining crypt length, number of cells per crypt, and number of cells proliferating. However, we did not directly measure the migration of cells up the crypt, for example by pulse-chase experiments, nor did we quantify cell shedding into the lumen, both of which are also important elements of turnover [15,45]. IECs originate from stem cells at the bottom of crypts, divide several times in the transit-amplifying zone (bottom half of crypts), after which they differentiate and finally are shed into the lumen due to crowding [13,45]. Nevertheless, *Setd7* deletion provides resistance to infection even in mice that completely lack an adaptive immune system. Thus, these results uncover an additional pathway to target in the design of therapies to treat helminth infection.

We also provide evidence that the developmental Hippo/YAP and Wnt/β-Catenin signaling pathways are important components of resistance to *T*. *muris* infection. Both Hippo and Wnt gene expression programs are induced upon infection and are mediated by SETD7. It would be particularly interesting to identify what cues drive these pathways during helminth infection, and whether the previously identified regulator of epithelial turnover, CXCL10, plays any role [15]. A potential alternative pathway that merges inflammation with regeneration is the recently described gp130-Src-YAP axis that is induced during intestinal inflammation [46]. Although this study did not identify a link with Wnt/β-Catenin, it remains to be seen if intestinal infection relies on this pathway. Nevertheless, our results show that SETD7-dependent regulation of Hippo and Wnt signaling plays a critical role in the development of resistance to intestinal helminth infection.

We used VP to test our hypothesis that YAP-TEAD mediated gene expression programs are important for *T*. *muris* resistance. Indeed, we find that VP treated animals become susceptible to *T*. *muris* compared to vehicle treated mice. However, we observed that VP treated animals also had increased T_H_1 cell-associated cytokines (IFN-γ) and reduced epithelial markers for resistance (TSLP and Muc5AC), indicating that the Hippo pathway may play a wider role. This is in line with a recent study identifying a role for YAP in goblet cell function [40]. In addition, it suggests that a YAP-TEAD mediated regenerative gene expression program is required to avoid a shift towards a T_H_1 response upon infection with *T*. *muris*.

In summary, we have identified a SETD7-dependent regulatory pathway in IECs that regulates immunity in the intestine. Modulation of SETD7 activity may provide a therapeutic strategy to improve anti-helminthic treatments independently of the innate and adaptive immune systems.

## Materials and Methods

### Mice and infection

*Villin*-Cre and *Rag1* ^*-/-*^ mice were obtained from Jackson Laboratories. *Vav-*Cre mice were obtained from T. Graf (Centre for Genomic Regulation, Barcelona, Spain). *Setd7* ^-/-^ and *Setd7* ^f/f^ mice were described previously [20,47]. We did not observe any physiological effects from Cre expression during homeostasis or infection. Animals were maintained in a specific-pathogen-free environment and tested negative for pathogens in routine screening. All experiments were carried out at the University of British Columbia following institutional guidelines. We used both males and females that were littermates and age matched (ranging from 7–15 weeks old) for all experiments. Isolation of *T*. *muris* eggs was carried out as described previously [24]. Mice were infected on day 0 with high dose (~200) or low dose (~35) of embryonated eggs by oral gavage, and parasite burdens were assessed microscopically on days 14, 21, 28, or 32 post-infection. Mice were infected with 200 *H*. *polygyrus bakeri* L3 larvae by oral gavage and parasite burdens were assessed microscopically on day 28. Liposome-encapsulated verteporfin (VP, Visudyne) was a kind gift by Novartis and was used at 50 mg/kg [42] by intraperitoneal injection at days -3, 0, 4, 7, 10, 13, 17, 20. Control mice were injected with the same volume of vehicle with the same schedule.

### Ethics statement

All experiments were performed according to protocols (A11-0290, A11-0329, A13-0010) approved by the University of British Columbia's Animal Care Committee and in direct accordance with The Canadian Council on Animal Care (CCAC) guidelines.

### Analysis of *T*. *muris* induced immunity

Mesenteric lymph node cells from *T*. *muris*-treated mice were isolated and single-cell suspensions were plated at 4 × 10^6^ per ml in the medium or in the presence of antibodies against CD3 (145-2C11) and CD28 (37.51); 1 μg ml^−1^ each; (eBioscience, San Diego, CA) or *T*. *muris* antigen (50 μg ml^-1^) for 72 h. Cytokine production from cell-free supernatants was determined by standard sandwich enzyme-linked immunosorbent assay (ELISA) using commercially available antibodies (eBioscience). *T*. *muris*-specific serum IgG1 and IgG2a levels were determined by ELISA on plates coated with *T*. *muris* Ag (5 μg ml^-1^). Total protein was isolated from fecal samples, resolved by sodium dodecyl sulfate- polyacrylamide gel electrophoresis, and immunoblotted using a rabbit anti-mouse RELM-β antibody (PeproTech, Rocky Hill, NJ).

### RNA extraction and qPCR

RNA was purified from whole intestine using mechanical disruption followed by TRIzol according to the manufacturer’s instructions, or from isolated IECs using RNeasy isolation kit (Qiagen). Reverse transcription using High Capacity cDNA Reverse Transcription kit (Applied Biosystems) was used to generate cDNA and qPCR was performed using SYBR green with primers from the Primer Bank (http://pga.mgh.harvard.edu/primerbank) using SYBR green chemistry on an ABI 7900 real-time PCR system (Applied Biosystems). Samples were normalized against *Actb* or *Gapdh* and are presented as fold over wild type, naïve, or relative to housekeeping gene as is indicated in figure legends.

### Tissue staining

Tissues were fixed in formalin and paraffin-embedded. Sections (5 μm) were stained with hematoxylin and eosin (H&E) or periodic acid-Schiff (PAS). Slides were analyzed on a Zeiss Axioplan2 microscope and images captured using a Qimaging Retiga EX CCD camera and Openlab 4.0.4 software (PerkinElmer). For immunofluorescence, 5 μm sections of paraformaldehyde-fixed, paraffin-embedded tissues were incubated with anti-Ki67 (SP6 clone, Sigma)) followed by Alexa568-conjugated goat-anti-rabbit and DAPI.

IEC turnover was calculated by Ki67+ cells / total cells (DAPI+) per crypt X 100. Of note, crypt length was under all conditions equally associated with total cells per crypt.

### Statistical analysis

Results represent the mean ± s.e.m. Statistical significance was determined by Student’s *t*-test or 1-way ANOVA with subsequent post hoc test.

## Supporting Information

S1 Fig No compensation of lysine methyltransferase expression in IECs from mice lacking *Setd7*. IECs were isolated from *Setd7* ^+/-^ and *Setd7* ^-/-^ mice and gene expression of indicated genes relative to the housekeeping gene *Actb* was determined by qPCR. n≥4.
(TIF)

S2 Fig Equal hatching *ex vivo*, and equal worm burden 12 days post infection. (A) Egg hatching was done in fecal extracts from indicated mice. n = 3. (B) Worm burden at day 12 post infection of indicated mice. Of note, at this point worms are very small and hard to distinguish, which leads to low numbers detectable. n = 5. (C) *Infg* and *Il13* expression in the proximal colon. Mice were killed at day 12 post infection. Gene expression is relative to infected control (*Setd7* ^+/-^) mice. n = 7.
(TIF)

S3 Fig Equal intestinal immune responses after *T*. *muris* infection in *Setd7* ^*ΔIEC*^ and *Setd7* ^*f/f*^ mice. (A) Gut gene expression of indicated genes and indicated mice at day 14 and day 21 post infection. Gene expression was calculated as fold over naive. n≥4. (B) Worm burden at day 12 post infection of indicated mice. Of note, at this point worms are very small and hard to distinguish, which leads to low numbers detectable. n = 7. (C) Expression of indicated genes and mice 12 days post infection. Gene expression is relative to infected control (*Setd7* ^f/f^) mice. n = 7 (D) IEC *Muc5ac* gene expression of naïve and *T*. *muris* infected mice at day 21 post infection. Fold over naive mice is shown. n≥5
(TIF)

S4 Fig Epithelial responses mediated by IEC-specific SETD7 during *T*. *muris* infection. (A) Ki67 and DAPI staining of ceacal sections after 14 and 21 days of *T*. *muris* infection. (B) Counts of number of nuclei per crypt as counted using DAPI staining as shown in (A). (C) Ratio between DAPI numbers (B) and crypt length (Fig 3B) showing that the ratio of those two parameters is equal, and independent of infection.
(TIF)

S5 Fig Chronic (low dose) *T*. *muris* infection in *Setd7* ^*ΔIEC*^ and *Setd7* ^*f/f*^ mice. (A) Worm burden at day 21 post infection with low dose (~35 eggs) *T*. *muris* of indicated mice. n = 4. (B) Gut gene expression of indicated genes and mice 21 days post infection. n = 4 (C) Gut gene expression of indicated genes and mice at 32 days post infection. (D) Serially diluted serum of infected mice was analyzed by ELISA to measure *T*. *muris-*specific IgG1.
(TIF)

S6 Fig IEC gene expression after *T*. *muris* infection. IEC gene expression of indicated genes of indicated mice after *T*. *muris* (*T*.*m*.) infection. n≥8 from at least 2 independent experiments.
(TIF)
