# Supplementary figures and images for: Intestinal Epithelial Cell-Intrinsic Deletion of Setd7 Identifies Role for Developmental Pathways in Immunity to Helminth Infection
Source: PLoS Pathog. 2016 Sep 6;12(9):e1005876. doi: 10.1371/journal.ppat.1005876 (PMC5012677; doi:10.1371/journal.ppat.1005876)

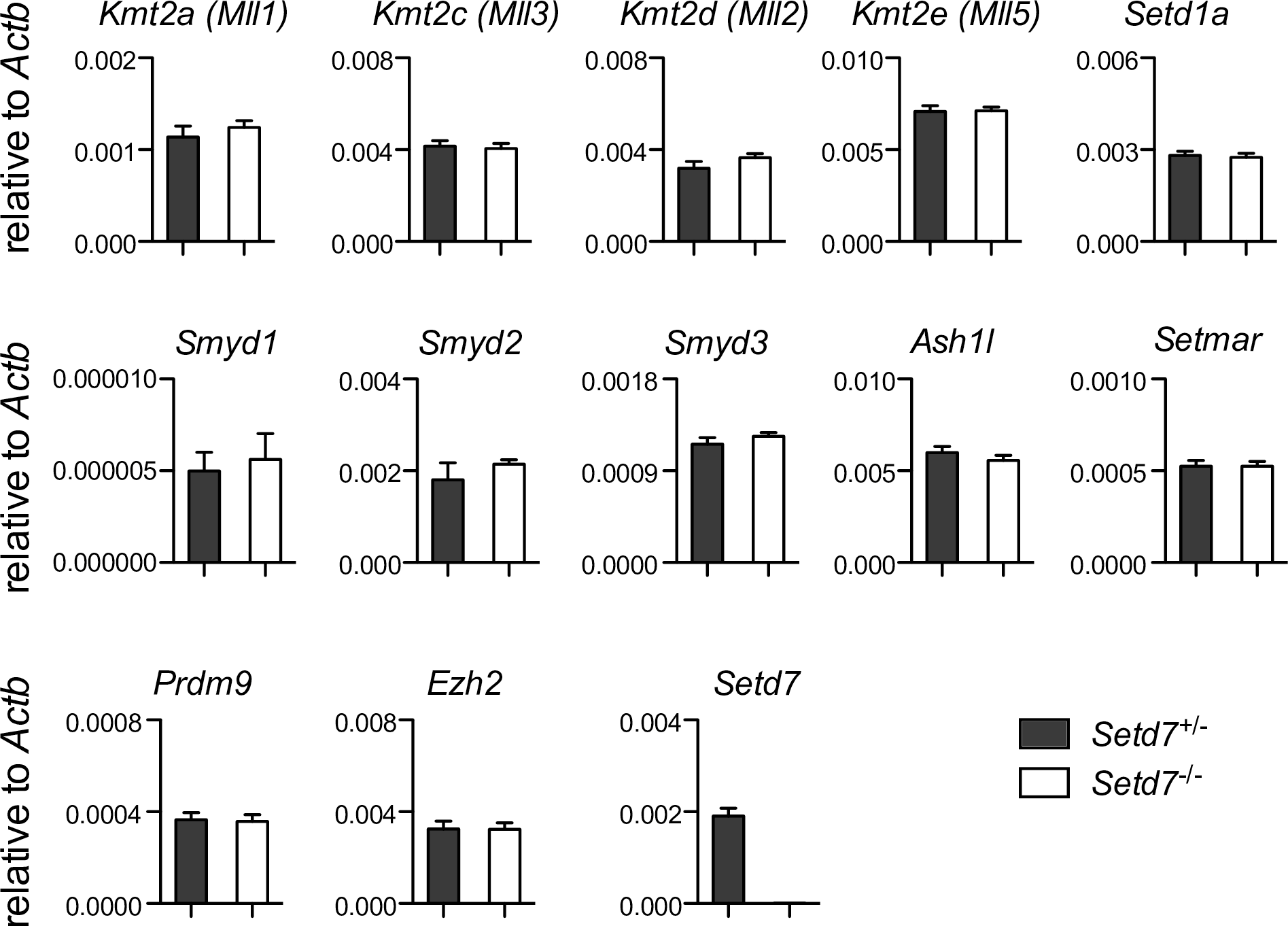

Supplement: S1 Fig — IECs were isolated from Setd7 +/- and Setd7 -/- mice and gene expression of indicated genes relative to the housekeeping gene Actb was determined by qPCR. n≥4. (TIF) [file ppat.1005876.s001.tif]

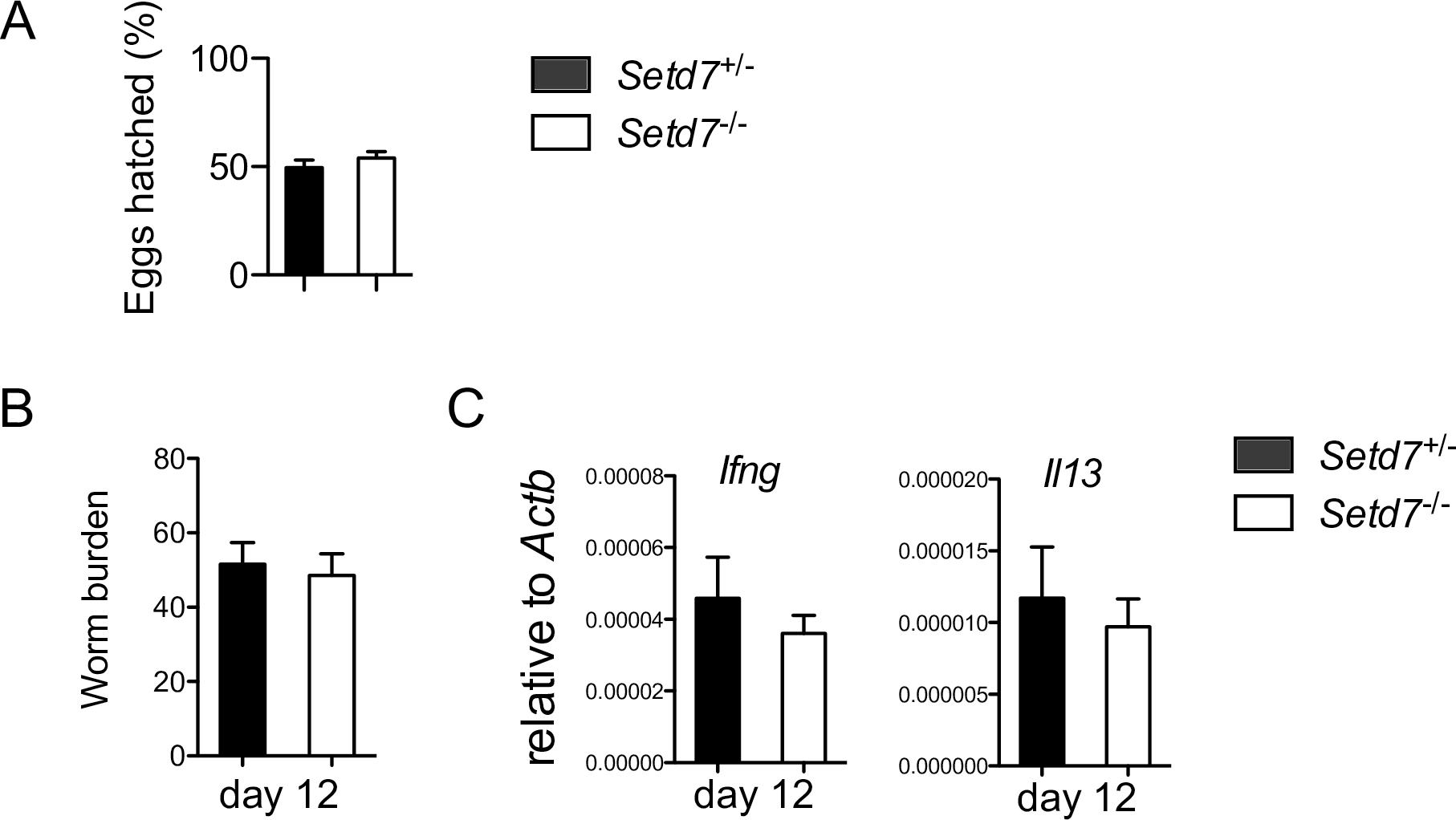

Supplement: S2 Fig — (A) Egg hatching was done in fecal extracts from indicated mice. n = 3. (B) Worm burden at day 12 post infection of indicated mice. Of note, at this point worms are very small and hard to distinguish, which leads to low numbers detectable. n = 5. (C) Infg and Il13 expression in the proximal colon. Mice were killed at day 12 post infection. Gene expression is relative to infected control (Setd7 +/-) mice. n = 7. (TIF) [file ppat.1005876.s002.tif]

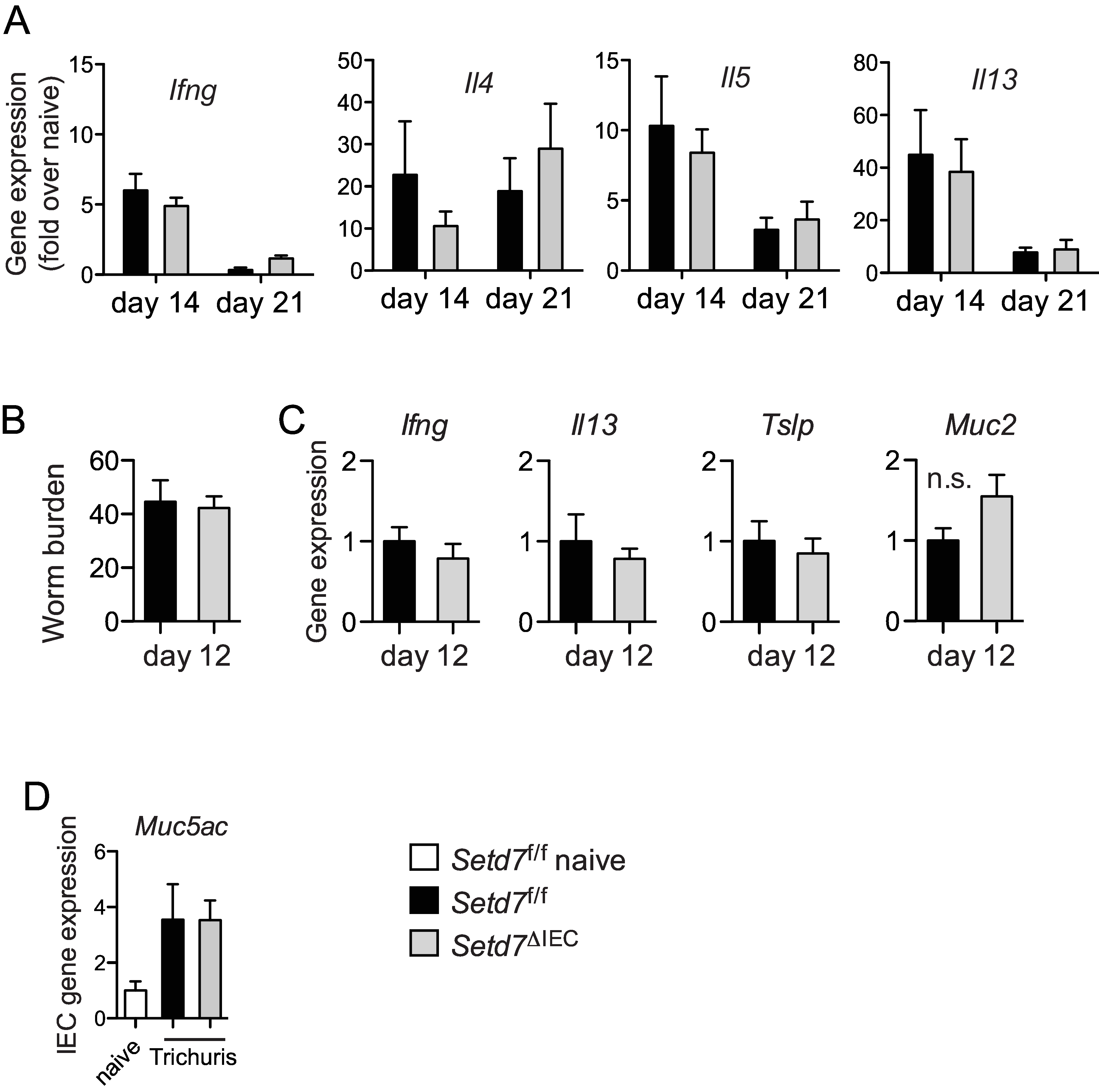

Supplement: S3 Fig — (A) Gut gene expression of indicated genes and indicated mice at day 14 and day 21 post infection. Gene expression was calculated as fold over naive. n≥4. (B) Worm burden at day 12 post infection of indicated mice. Of note, at this point worms are very small and hard to distinguish, which leads to low numbers detectable. n = 7. (C) Expression of indicated genes and mice 12 days post infection. Gene expression is relative to infected control (Setd7 f/f) mice. n = 7 (D) IEC Muc5ac gene expression of naïve and T. muris infected mice at day 21 post infection. Fold over naive mice is shown. n≥5 (TIF) [file ppat.1005876.s003.tif]

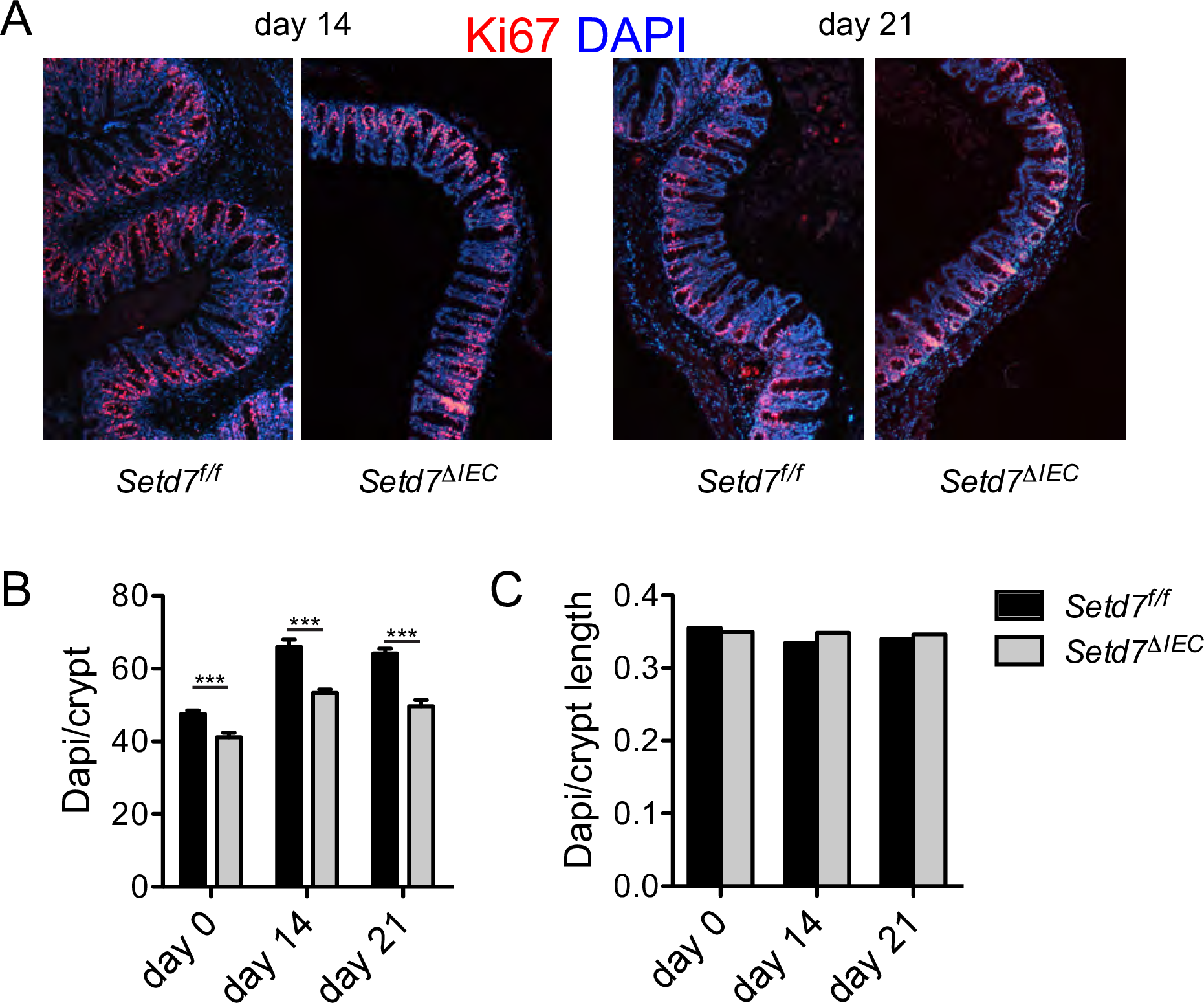

Supplement: S4 Fig — (A) Ki67 and DAPI staining of ceacal sections after 14 and 21 days of T. muris infection. (B) Counts of number of nuclei per crypt as counted using DAPI staining as shown in (A). (C) Ratio between DAPI numbers (B) and crypt length (Fig 3B) showing that the ratio of those two parameters is equal, and independent of infection. (TIF) [file ppat.1005876.s004.tif]

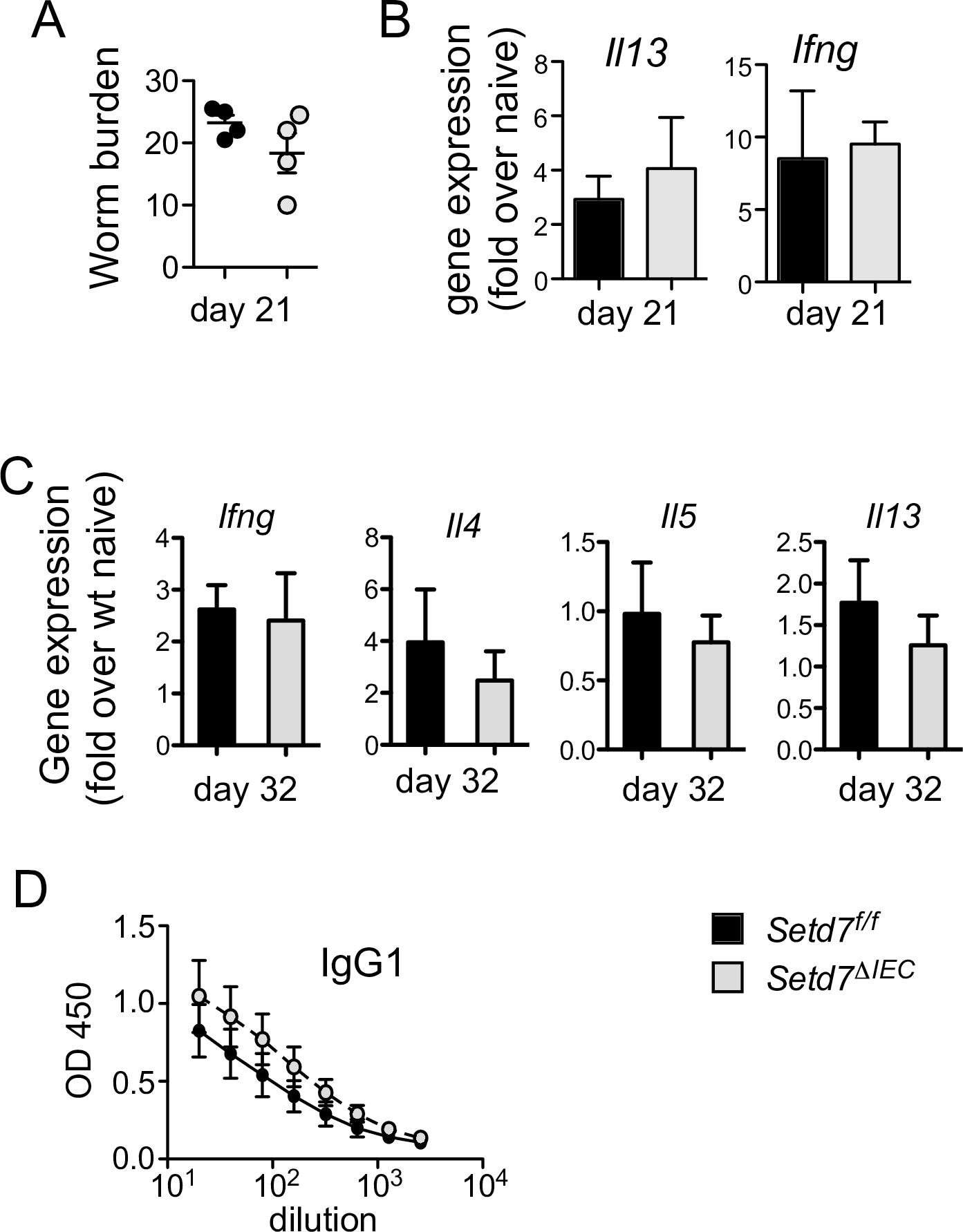

Supplement: S5 Fig — (A) Worm burden at day 21 post infection with low dose (~35 eggs) T. muris of indicated mice. n = 4. (B) Gut gene expression of indicated genes and mice 21 days post infection. n = 4 (C) Gut gene expression of indicated genes and mice at 32 days post infection. (D) Serially diluted serum of infected mice was analyzed by ELISA to measure T. muris-specific IgG1. (TIF) [file ppat.1005876.s005.tif]

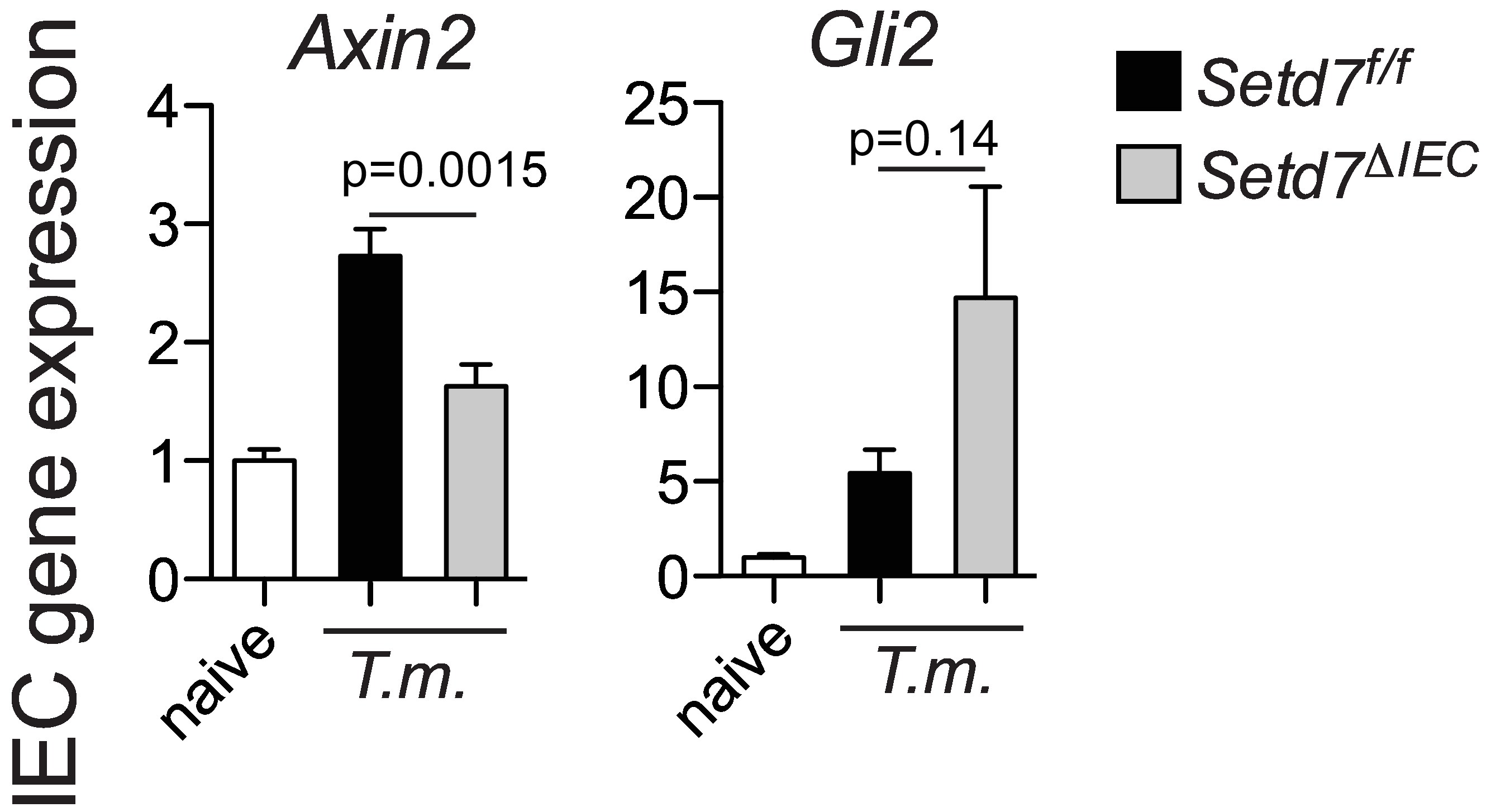

Supplement: S6 Fig — IEC gene expression of indicated genes of indicated mice after T. muris (T.m.) infection. n≥8 from at least 2 independent experiments. (TIF) [file ppat.1005876.s006.tif]
